# Employer Perceptions and Responses to an Aging Workforce: Differences by Industry, Occupation, and Size

**DOI:** 10.1093/geroni/igaa057.2941

**Published:** 2020-12-16

**Authors:** Robert Clark

**Affiliations:** North Carolina State University, Raleigh, North Carolina, United States

## Abstract

Employers are facing significant demographic changes in their workforce as the US population continues to age and older workers seek to delay retirement until later ages. The challenges associated with workforce aging vary substantially across the economy and vary by industry, occupation, and size of the organization. This analysis highlights the findings from employer surveys and workshops of senior HR leaders. The article summarizes the findings from our three-year research project including whether employers perceive that workforce aging is a challenge to their organization and if so, what changes in compensation and working conditions are being made to accommodate the changes in age structure

